# Electrothermal catalysis: paradigm shift from monolithic Joule heating to interparticle electrical promotion

**DOI:** 10.1093/nsr/nwag167

**Published:** 2026-03-17

**Authors:** Xueyi Mei, Yexin Zhang, Zhaoliang Zhang, Jian Zhang

**Affiliations:** Ningbo Institute of Materials Technology and Engineering, Chinese Academy of Sciences, Ningbo 315201, China; Ningbo Institute of Materials Technology and Engineering, Chinese Academy of Sciences, Ningbo 315201, China; School of Chemistry and Chemical Engineering, University of Jinan, Shandong Key Laboratory of Green and Low Carbon Recycling and Application of Rare Earth Materials, Jinan 250022, China; Ningbo Institute of Materials Technology and Engineering, Chinese Academy of Sciences, Ningbo 315201, China; University of Chinese Academy of Sciences, Beijing 100049, China

**Keywords:** electrothermal catalysis, conductive catalyst, Joule heating, electrical promotion, carbon neutrality

## Abstract

Conventional thermal catalysis, dependent on carbon-intensive fossil fuels, faces pressing sustainability challenges. As an alternative relying on renewable electricity, electrothermal catalysis utilizing catalyst Joule heating enables decarbonized chemical processes and is advancing rapidly under carbon-neutrality mandates. Here, we clarify the fundamental characteristics and scope of electrothermal catalysis, especially highlighting advances in our own works. Accordingly, a paradigm shift from monolithic Joule heating to interparticle electrical promotion is proposed. Monolithic catalyst architectures employ the Joule heating of monolithic supports for compact and efficient heating, whereas interparticle counterparts leverage both Joule heating and electrical promotion between conductive catalyst particles, not only substantially enhancing the intrinsic activity of catalysts, but also drastically reducing energy costs and carbon footprints for the sustainable electrification of chemical processes. Ultimately, the perspectives and challenges of interparticle electrical promotion are highlighted and analysed to advance this transformative shift toward a viable industrial process.

## Teaser

Catalyst interparticle electrical promotion beyond simple Joule heating offers a new way to power chemical reactions, drastically enhancing catalytic performance while minimizing the energy cost and carbon footprint.

## INTRODUCTION

Catalysis is one of the pillars of the chemical and energy industries, and >80% of industrial processes rely on catalysts [1–3]. Amongst them, heterogeneous catalysis utilizing solid catalysts dominates large-scale industrial processes [3] and also plays pivotal roles in environmental remediation [4] and carbon neutrality [5]. Most catalytic processes, involving the breaking of chemical bonds and the formation of new ones, are energy-intensive, occurring at high temperatures [6]. For instance, the ammonia synthesis in the Haber–Bosch process usually operates at temperatures of >400°C [7] and the steam reforming of methane (SRM) for hydrogen production requires high temperatures of 600°C–1000°C [8]. However, the maintenance of high-temperature reactions predominantly depends on the combustion of fossil fuels, leading to significant levels of CO_2_ emissions. As exemplified above, 1 tonne of ammonia production would generate ∼1.1 tonnes of CO_2_ [9], while the industrial SRM process releases 9 kg of CO_2_ per kg of H_2_ [10]. Therefore, carbon-intensive thermal catalysis goes against the global pursuance of carbon-reduction efforts currently underway.

The electrification of catalytic processes, powered directly by electricity, could shift the reliance on fossil fuels to renewable (green) energy such as solar and wind, thereby enabling the establishment of cleaner chemical processes with significantly reduced CO_2_ emissions [11,12]. This is intriguing because the share of electricity production from renewables grew to 30.24% in 2023 [13] and electrification can utilize the renewables on-site that are difficult to integrate into the power grid. Until now, several electrification methods for catalysis have been developed, including electrocatalysis, plasma, induction heating, microwave/radio frequency heating and direct resistive heating [14,15]. Amongst them, direct resistive heating by passing a high-power electrical current through a material with low resistivity, resulting in heat generation from resistance, is more appealing due to its higher technology readiness levels and retrofitting for existing facilities [15]. Furthermore, the conductive materials employed are integral parts of the catalysts or are in intimate contact with them, different from indirect resistive heating (external heating) via an electrical oven [16].

The direct resistive heating method for catalysis has been reported in numerous works, yet the terminology used is not consistent. Besides the term ‘direct resistive heating catalysis’ [17,18], others include ‘Joule heating catalysis’ [19–21], ‘electric internal heating catalysis’ [22], ‘electric-field-enhanced catalysis’ [23], ‘electro-injection-enhanced catalysis’ [24], ‘Joule-heated interfacial catalysis’ [25], ‘current-assisted catalysis’ [26], ‘electrothermal catalysis’ [27,28], etc. In fact, the electrification process encompasses not only Joule heating, which supplies thermal energy for reactions, but also electrical promotion that enhances the intrinsic activity of catalysts. The word ‘electrothermal’ aptly captures both thermal and electrical effects in an integrated manner. Therefore, we have chosen ‘electrothermal catalysis’ as the uniform terminology. Although this term could be literally and broadly associated with other forms of electricity-powered catalysis (e.g. microwave, induction, plasma, electrocatalysis or even photothermal catalysis), the crucial characteristic of the definition is its unique mode of energy conversion: Joule (resistive) heating within the conductive catalyst itself, which distinguishes it from other electrified catalysis processes.

Electrothermal catalysis has undergone rapid growth and some reviews on the subject have been made. Stankiewicz *et al*. [14] classified resistance-heated reactors as one type of electricity-based reactors, highlighting their advantages in uniform heat transfer. Mallapragada *et al*. [15] proposed direct resistive heating as a potential approach for decarbonization in the chemical industry. Wang *et al*. [29] provided an overview of the application of electrothermal catalysis along with catalytic mechanisms and reactor designs. Zheng *et al*. [30] concluded that, in comparison with other electrification approaches, resistance heating represents the most promising method for industrialization, given its advantages in materials, reactor design and temperature flexibility. Griffin *et al*. [31] contributed a detailed overview of the Joule-heating reactor design criteria for chemical and advanced materials synthesis. Cui *et al*. [32] put forward the Joule-heating catalysis concept, elucidating its basic principle, scientific connotation and action mechanism. Zhu *et al*. [23] defined electrothermal catalysis as a type of electrical heating technique that occurs through Joule heating. They also categorized the conductors and devices used in the field of catalytic oxidation of pollutants and pointed out the coexisting thermal and electrical effects. By reviewing historical developments, Liu *et al*. [33] proposed that the current-assisted catalytic strategy heralds the electrification era of catalysts. Despite these existing comprehensive reviews, the boundary of electrothermal catalysis remains indistinct and it is frequently confused with other forms of electrified catalysis, such as plasma catalysis and electric-field-assisted catalysis. Furthermore, while most studies focus on utilizing Joule heating within catalysts, less attention has been paid to the electrical promotion of the intrinsic activity of catalysts associated with catalyst architectures.

In this review, we clarify the unique characteristics of electrothermal catalysis with consistent terminology and explicit scope, distinguishing it from other electrified catalysis methods including plasma catalysis and electric-field-assisted catalysis. Specifically, the catalyst architectures are categorized into two distinct groups based on the literature and especially our own works. One uses conductive monolithic materials, such as metal-and carbon-based materials as well as conductive ceramics, as catalyst supports to utilize their Joule heating for reactions. The other constitutes interparticle conductive catalysts such as powdered semiconductors of metal oxides. In addition to interparticle Joule heating, the interparticle catalyst architectures present amplified electrical promotion of the intrinsic activity of the catalyst. Accordingly, we propose a paradigm shift in electrothermal catalysis—from monolithic Joule heating to interparticle electrical promotion—and address the future prospects and challenges of this shift.

## CLARIFYING THE SCOPE OF ELECTROTHERMAL CATALYSIS

Plasma catalysis and electric-field-assisted catalysis share similarities in reactor configuration with electrothermal catalysis. As depicted in Table 1, catalysts are connected to electrical power sources, allowing voltages to be applied to them for reactions. The ambiguous boundaries between them lead to frequent confusion. Therefore, we present their discrepancies in characteristics in Table 1, mainly concerning the electrical properties of catalysts: (i) plasma catalysis employs insulating catalysts with high dielectric constants [34], (ii) electric-field-assisted catalysis utilizes weakly conductive catalysts [35] and (iii) electrothermal catalysis requires relatively highly conductive catalysts [29]. Correspondingly, the operating voltages decrease significantly with increasing catalyst conductivity: (i) plasma catalysis necessitates kilovolt-range voltages to generate energetic electrons for reactant/catalyst activation [34], (ii) electric-field-assisted catalysis operates at hundreds of volts with milliampere-level currents to assist reactions [35], and (iii) electrothermal catalysis employs low voltages (several to dozens of volts) with large currents (amperes) to induce Joule heating [29]. A further critical distinction is the need for external heating: (i) electrothermal catalysis eliminates the need for external heating [29], (ii) plasma catalysis requires external heating in some cases [36], and (iii) electric-field-assisted catalysis typically relies on external heating [35]. Thus, electrothermal catalysis is uniquely characterized by:

- conductive catalysts;
- Joule heating induced by high current (amperes) under low voltage (several to dozens of volts);
- independence from external heating.

**Table 1.** Comparison of characteristics between plasma catalysis, electric-field-assisted catalysis and electrothermal catalysis.

Characteristics	Plasma catalysis	Electric-field-assisted catalysis	Electrothermal catalysis
Reactor diagram			
	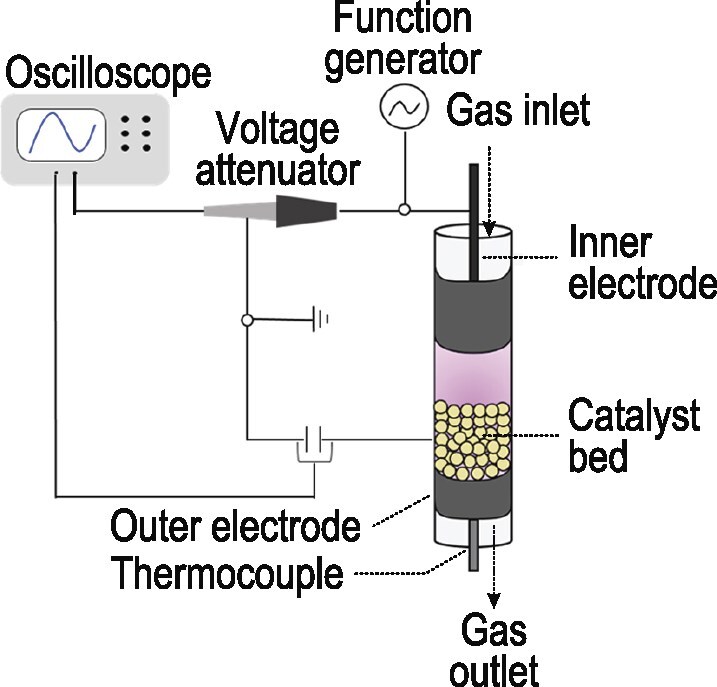 Ref. [36]	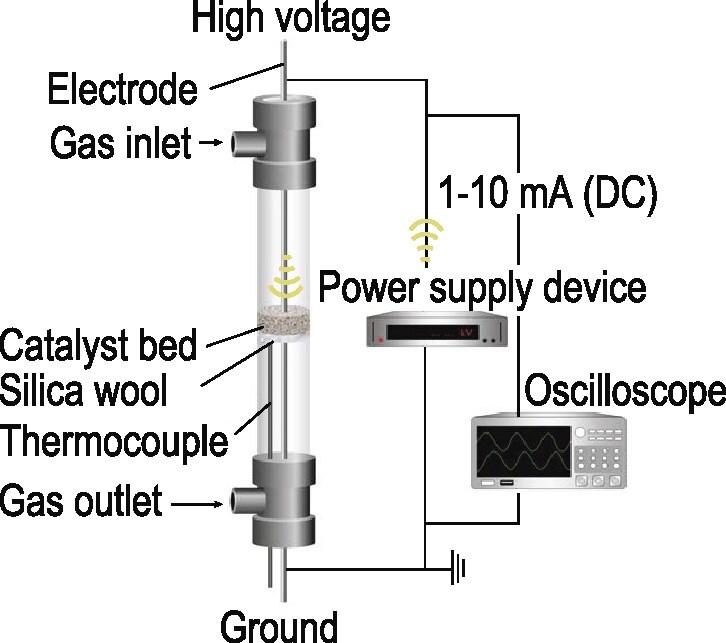 Ref. [35]	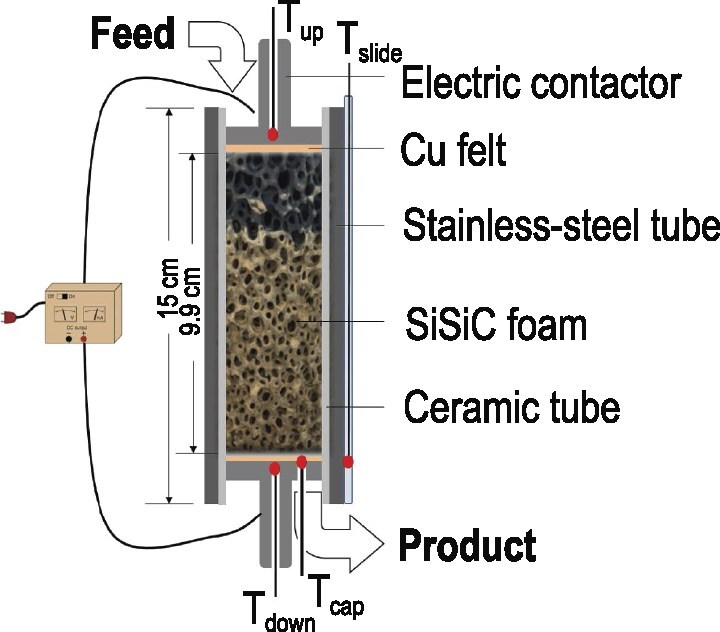 Ref. [37]
Electric conductivity of catalysts	Insulation with high dielectric constants	Weak conductivity	Good conductivity
Voltage	>1 kV	Hundreds of volts	Several to dozens of volts
Current	No current	Low current in milliamperes	High current in amperes
External heating	Partial dependence on external heating	Dependence on external heating	Independence from external heating

Adapted with permission from Refs [36] (Copyright 2023, American Chemical Society), [35] (Copyright 2021, Royal Society of Chemistry) and [37] (Copyright 2023, Elsevier).

Based on this defined scope and its characteristics, we have selected studies for inclusion in this review that focus specifically on electrothermal catalysis.

## ELECTROTHERMAL CATALYSIS USING MONOLITHIC CONDUCTIVE SUPPORTS

### Metallic supports

**Figure 1.**
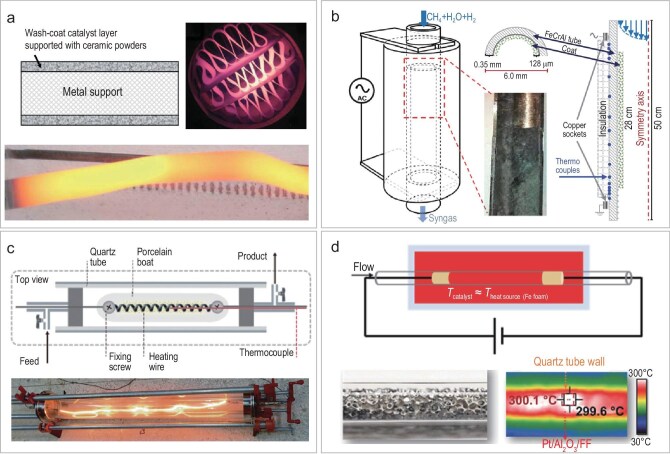
Metallic supports for electrothermal catalysis. (a) Plate-type alloy coated with Ni/nickel aluminate catalysts. Adapted with permission from Ref. [41] (Copyright 2009, Elsevier). (b) FeCrAl-alloy tube coated with Ni-supported Zr-based catalysts. Adapted with permission from Ref. [16] (Copyright 2019, AAAS). (c) FeCrAl wire. Adapted with permission from Ref. [46] (Copyright 2024, Springer Nature). (d) Fe foam coated with Pt/Al_2_O_3_ catalysts. Adapted with permission from Ref. [47] (Copyright 2023, Springer Nature).

The commonly accessible monolithic conductive materials, which consist of metal, carbon or conductive ceramics, are employed as catalyst supports for electrothermal catalysis due to their ability to generate stable currents and Joule heating when subjected to low voltages. The Joule-heating supports reported are summarized in Table 2. Amongst them, metallic supports with various shapes, such as steel wool, Ni foam, wire mesh and alloy wire, are preferred owing to their excellent conductivity and machinability. Kameyama’s group developed a series of electrified reactors by using plate-type alloys (including CrAl [38], NiCr [39], FeCr [40], FeCrNi [41–43]) as Joule-heating supports with differently coated catalysts (Fig. 1a). These compact reactors demonstrate excellent reforming performance for hydrogen production, characterized by a short startup time and highly efficient heat transfer. Wismann *et al*. [16,44,45] employed FeCrAl-alloy tubes as Ni-catalyst supports for SRM (Fig. 1b), creating intimate contact between the electric heat source and the reaction sites to drive the reaction close to thermal equilibrium. If this flexible and compact reactor that is potentially 100 times smaller in scale than current reformer platforms were to be globally adopted, then CO_2_ emissions would be reduced by 1%. Ma *et al*. [46] directly utilized the Joule heating of FeCrAl wire (Fig. 1c), which also provides catalytically active sites, to reform plastics with CO_2_ into syngas at high yields and carbon balance, addressing environmental issues in a circular economy. This process is energy-saving without heat conduction and heat exchange, and its sustainability merit could be further enhanced by using photovoltaic power under solar irradiation through a life-cycle assessment. Wang *et al*. [47] selected Fe foam with a 3D network structure as the Joule-heating support, coating it with Pt/Al_2_O_3_ catalyst for methylcyclohexane dehydrogenation (Fig. 1d). Electrification can efficiently reduce heat- and mass-transfer limitations and prominently boost dehydrogenation performance, with a hydrogen-evolution rate that is two to five times higher than those of the reported Pt-based catalysts under conventional thermal catalysis. Balakotaiah *et al*. [48] proposed that parallel wire configurations could offer the greatest flexibility for endothermic reactions in thermal efficiency with uniform current and heat flux, based on the numerical simulation of various modular reactor configurations.

**Table 2.** Summary of monolithic conductive supports reported for electrothermal catalysis.

Monolithic conductive support	Catalyst coating	Reaction	Features	Refs
Stainless steel screens	Ni/Al_2_O_3_	Steam reforming of methane	• Compact workshop-assembled units • Potential for low maintenance costs	[49]
Steel wool	No coating	Dry reforming of methane	• Dramatic effect on activity • Using green energy available	[50]
Flat Hastelloy C-276 strip	Pt/TiO_2_ and Fe_2_O_3_	SO_3_ decomposition	• Reducing heat loss	[17]
Ti wire	Titanium oxide	Three-way catalytic reaction	• Fast heating • Promptly eliminating hydrocarbons in cold-start exhaust	[51]
Pt film	No coating	Oxidation of CO	• Pulsed activation in heterogenous catalysis	[52]
Pt film	Pt/Al_2_O_3_	Oxidation of CO	• Providing fast, well-controlled temperature pulses • 40× rate enhancement • Power input lower than that for steady-state operation	[53]
CrAl-alloy plate	Ni/Al_2_O_3_	Steam reforming of methane	• High heat-transfer coefficient • Good transverse gas-temperature distribution • Short startup time • High activity and stability	[38]
NiCr-alloy plate	Ni/NiAl_2_O_4_/γ-Al_2_O_3_	Steam reforming of methane	• No unfavorable effect over catalyst reactivity	[39]
FeCr-alloy plate	Ni/Al_2_O_3_	Steam reforming of methane	• Short startup time	[40]
FeCrNi-alloy plate	Ni/nickel aluminate layer	Steam reforming of methane	• Excellent reactivity and durability	[41]
FeCrNi-alloy plate	Ru/Al_2_O_3_	Steam reforming of kerosene	• Short startup time	[42]
FeCrNi-alloy plate	CeNi/Al_2_O_3_	Steam reforming of ethanol	• Producing bioethanol at low cost	[43]
FeCrAl alloy	LaNi_0.95_Ru_0.05_O_3_	Combined dry and steam reforming	• Proof of concept for electrically heated dry reforming of methane	[54]
FeCrAl porcupine coil	MnO*_x_*/ZSM-5	Ethane dehydrogenation	• Higher gas–catalyst contact area and mass transfer • Flexible, cost-effective and scalable heating element	[55]
FeCrAl wire	Ni/MgO–CeO_2_	Dry reforming of methane	• Rapid temperature response • Enhancing heat transfer • High activity and superior coking resistance	[56]
FeCrAl-alloy wire	Pd/Al_2_O_3_	Oxidation of ethylene	• Simple and efficient Joule-heat-ignition reactor	[19]
FeCrAl-alloy wire	Mn/Al_2_O_3_	Oxidation of CO and toluene	• Rapid ignition of reactions with simplified devices	[57]
Square alloy wire mesh	V_2_O_5_–WO_3_/TiO_2_	Selective catalytic reduction by ammonia	• Superior performance • High resistance to H_2_O and NH_4_HSO_4_ poisoning	[58]
FeCrAl-alloy tube	Ni-supported Zr-based wash coat	Steam reforming of methane	• Flexible and compact heat generation • Intimate contact between electric heat sources and reaction sites • Driving reactions close to thermal equilibrium • Increasing catalyst utilization • Limiting unwanted byproduct formation • Compact reactor design • Predicting nearly 1% of all CO_2_ emissions reduction if used globally	[16]
FeCrAl-alloy tube	Ni-supported Zr-based wash coat	Steam reforming of methane	• Resolving limiting thermal conductivity across catalysts	[44]
FeCrAl-alloy tube	Ni-supported Zr-based wash coat	Steam reforming of methane	• Fast startup or shutdown • Minimal thermal gradients • Closing to thermodynamic limits for carbon formation • Scalability to industrial conditions	[45]
NiCrAl-alloy foam	Ru	Decomposition of ammonia	• Minimizing heat-transfer scale • Low reactor volume • High efficiency and power density	[59]
FeCrAl-alloy wire	Pt or Pd nanoparticles	Oxidation of VOCs	• Requirement of small equipment • High-efficiency energy utilization • Self-heating • Rapid response • High activity and stability	[60]
FeCrAl-alloy wire	No coating	Dry reforming of plastics	• Fast heating • Avoiding heat conduction and heat exchange • Excellent coke resistance • Sustainability merit using photovoltaic power under solar irradiation through a life-cycle assessment	[46]
FeCrAl-alloy monolith	Ni/MgO	Dry reforming of methane	• Rapid and uniform heating • Energy-concentrated and ultra-compact reactor system • Syngas production capacity tens of times higher than those of other studies	[61]
FeCr-alloy monolith	NiO/MgO/γ–Al_2_O_3_	Steam reforming of tars	• Rapid heating • Controlling coke formation	[62]
FeCrAl strip	Pd	Oxidation of toluene	• Reducing energy consumption by >50 times • Convenient electron transfer between electric power supply and reaction active sites • Enhanced electron transfer of lattice oxygen • Rapid ignition and cool-down response • Good reusability, long-term stability and high electricity-to-heat efficiency	[27]
Ni foam	*δ*-MnO_2_	Oxidation of formaldehyde	• High activity, water resistance and durability • Enhanced release of lattice oxygen	[63]
Ni foam	CuO–ZnO	Methanol decomposition	• Higher conversion and lower energy consumption • Promoting the migration of oxygen and redox of Cu/Zn species	[64]
Ni foam	Pd/NiO	Oxidation of VOCs	• Small size and rapid response • High catalytic activity and safety	[65]
Ni foam	Pt/CeO_2_	Oxidation of toluene	• Minimal energy consumption (87%) • Ultra-low gas resistance • Short response time • Simplified setup design	[20]
Ni foam	*δ*-MnO_2_ decorated layered double oxides	Oxidation of *n*-heptane	• Simple equipment • Convenient operation • Fast response • High energy-utilization rate • Good stability	[28]
Ni foam	MnO_2_/TiO_2_	Oxidation of acetone	• Low energy consumption • Improving low-temperature catalytic activity • Electric effect enhancing conversion of lattice oxygen to surface-adsorbed oxygen species and promoting the oxidation of Mn oxides	[66]
Ni foam	Ni, RuNi, Co or RuCo/Ni-layered double hydroxides	CO_2_ methanation	• Uniform temperature distribution • Enhanced catalytic activity and anti-poisoning ability • Accelerating the reduction of Ni^2+^ • Electron transfer between the catalytic sites induced by the external voltage	[22]
Ni foam	LaNi/Ni-layered double hydroxides	CO_2_ methanation	• Superior reactivity to most reported Ni-based catalysts • High sulfur resistance • Decreased temperature gradient	[67]
Ni foam	Ce–OMS-2	Oxidation of toluene	• Improving reactivity and moisture resistance • Flexible, compact and efficient thermal control	[68]
Ni foam	MnO*_x_*–CeO_2_	Oxidation of chlorobenzene	• Low-temperature activity, long-term stability and water tolerance • Less toxic byproducts • Reducing energy consumption by >93% • Rapid temperature response • Boosting the activation of lattice oxygen	[69]
Ni foam	Ni/Al_2_O_3_	Dry reforming of methane	• Stable running for 50 h • Enabling syngas and carbon nanotube co-production • Reducing syngas costs and carbon emissions	[70]
Fe foam	Pt/Al_2_O_3_	Methylcyclohexane dehydrogenation	• H_2_-evolution rates two to five times higher than those of thermal catalysis processes • High mass- and heat-transfer ability • Low thermal resistance and heat capacity • Fast temperature response	[47]
3D structured carbon	Ni	Ammonia decomposition	• Highly efficient carbon-based Joule heaters embedded with catalytic nanoparticles • Low energy consumption	[71]
3D structured carbon	Ru	Ammonia decomposition	• Upcycling of mixed polyolefin waste into 3D structured carbons • Enhanced NH_3_ conversion • Decreasing greenhouse gas and life-cycle energy consumption	[72]
Carbon cloth	MnO_2_	Oxidation of formaldehyde	• Long-term formaldehyde-removal efficiency • Close-contacted interface allowing efficient heat transfer • Electrically generating O^2−^ species	[73]
Carbon cloth	MnO_2_	Oxidation of formaldehyde	• Electric field increasing the electron state density of Mn atoms • Electric field promoting the generation of •OH and •O_2_^−^ • Electric field promoting the degradation of intermediate products	[23]
Carbon membrane	*α*-MnO_2_	Oxidation of formaldehyde	• Developing an air cleaner by integrating MnO_2_ catalysts with heating equipment • Generated heat drives movement of the gas and forms thermal circulation	[74]
Mesoporous-carbon monoliths	Ag–Co_3_O_4_	Oxidation of formaldehyde	• Precise heating process • 87% decline in energy consumption	[75]
Wood carbon monoliths	No coating	Methane pyrolysis	• High profit predicted by techno-economic analysis	[61]
Porous carbon paper	No coating Rh nanoparticles	Methane pyrolysis Ammonia synthesis	• Non-equilibrium, continuous synthesis technique using pulsed heating and quenching • Precise control over the heating process leads to high selectivity • Preventing catalyst sintering	[76]
Carbon-fiber paper	PtNi/SiO_2_	Dry reforming of methane	• Pulse Joule heating creating *in situ* catalyst regeneration • Suppressing coke formation, sintering and phase segregation	[77]
Carbon-fiber paper	Ni/Al_2_O_3_ Ni/SiO_2_	CO_2_ hydrogenation	• Pulse Joule heating facilitating CO desorption and suppressing the subsequent deep deoxygenation and hydrogenation to CH_4_	[78]
Carbon felt	COF–SO_3_H	Esterification	• Intimate contact between the electric heat source and the catalyst • Efficient, localized Joule heating directly at catalytic sites • Minimizing thermal losses • Allowing precise control over reaction interfaces	[25]
(La_0.80_Sr_0.20_)_0.95_FeO_3_/ Gd_0.1_Ce_0.9_O_2_ ceramic	MnO_2_	Oxidation of CO	• Flexibility to control reactor temperatures	[21]
SiC ceramic	Ni/silica–mullite	Steam and dry reforming reactions	• Energy consumption for H_2_ production comparable to those of modern electrolysers • Reversing the flow of heat from the inside of the catalytic bed to the outside	[79]
SiSiC foam	Ni/CeO_2_/Al_2_O_3_	Dry reforming of methane	• Approaching thermodynamic equilibrium • Energy consumption close to the theoretical value • Energy consumption equal to ∼1/4 of that for microwave heating	[80]
SiSiC foam	Rh/Al_2_O_3_	Steam reforming of methane	• Methane conversions approaching equilibrium • High energy efficiency of 61% • Low-carbon hydrogen production	[30]
SiSiC foam	Rh/Al_2_O_3_	Steam reforming of methane	• Low specific energy demands at high space velocities • Mathematical model for preliminary scale-up calculations	[81]
SiSiC foam	Rh/Al_2_O_3_	Reverse water–gas shift Dry reforming of methane	• Remarkably low specific energy demand for CO_2_ vaporization • Compact small-scale reactors	[37]
SiSiC foam	Ni/Al_2_O_3_	Dry reforming of methane	• Energy saving with low reactor wall temperature • Volumetric heating with uniform temperature distribution • Higher activity than external heating	[82]
SiC foam	Ni–CHA	Dry reforming of methane	• Superior space–time yield of syngas • Remarkable stability of 330 h • No coke deposition	[83]

VOC, volatile organic compound.

Metallic materials serving as Joule-heating supports for electrothermal catalysis can achieve a compact reactor design, minimizing heat-transfer and temperature gradients, with fast startup or shutdown and low energy consumption. However, some limitations of metallic supports should not be overlooked. First, the super-high conductivity of metallic supports typically implies relatively high currents, ranging from tens to hundreds of amperes [50,62,66], to generate pronounced Joule heating, which tends to result in significant electric energy loss as it flows through the entire circuits, particularly at connection points with high resistance. Second, the non-porous structure and extremely low surface area of metallic supports restrict the coating of catalysts. To attain higher coating amounts, the catalyst layer is generally made thicker, elevating temperature gradients and limiting mass transfer [44]. Third, metallic supports generally mismatch catalyst coatings in thermal expansion, destroying the adhesion between them at high temperatures [40]. Finally, metallic supports are susceptible to corrosive, oxidative or hydrothermal atmospheres, undermining their stability [21,41].

### Monolithic carbon supports

**Figure 2.**
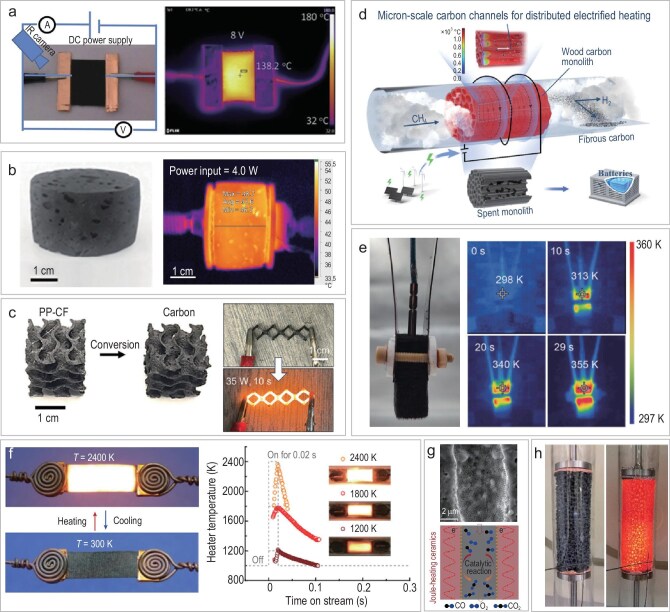
Carbon-based and conductive ceramic supports for electrothermal catalysis. (a) Carbon cloth coated with MnO_2_. Adapted with permission from Ref. [73] (Copyright 2019, Elsevier). (b) Mesoporous-carbon monolith coated with Ag–Co_3_O_4_ catalysts. Adapted with permission from Ref. [75] (Copyright 2020, Elsevier). (c) Structured carbon Joule heater from 3D-printed plastic waste. Adapted with permission from Ref. [71] (Copyright 2025, John Wiley and Sons). (d) Wood carbon monolith with micron-scale channels. Adapted with permission from Ref. [61] (Copyright 2024, Springer Nature). (e) Carbon felt coated with COF–SO_3_H catalysts. Adapted with permission from Ref. [25] (Copyright 2024, John Wiley & Sons). (f) Porous carbon paper. Adapted with permission from Ref. [76] (Copyright 2022, Springer Nature). (g) (La_0.80_Sr_0.20_)_0.95_FeO_3_/Gd_0.1_Ce_0.9_O_2_ microchannel ceramics coated with MnO_2_ catalysts. Adapted with permission from Ref. [21] (Copyright 2022, Springer Nature). (h) SiSiC foam coated with Rh/Al_2_O_3_ catalysts. Adapted with permission from Ref. [37] (Copyright 2023, Elsevier).

Carbon-based materials are ideal candidates to substitute for metallic materials as Joule-heating supports in electrothermal catalysis, thanks to several advantages, including porous structures with high surface areas, ease of modification, low coefficient of thermal expansion and suitable conductivity for Joule heating. Zou *et al*. [73] utilized the Joule heating of a carbon-cloth-based MnO_2_ catalyst to achieve long-term formaldehyde removal (Fig. 2a). When a voltage of 8 V was input, the surface temperature of the composite rapidly rose to 138°C within 20 s, enabling swift *in situ* regeneration of the catalyst during the reaction. Wang *et al*. [75] fabricated a mesoporous-carbon monolith with coated Ag/Co_3_O_4_ catalysts for the catalytic removal of formaldehyde (Fig. 2b). The precise Joule heating of the monolith is directly delivered to the catalyst, without the requirement to heat the largely excessive air and surroundings, drastically reducing the energy consumption by 87%. Qiang’s group [71,72] transformed 3D-printed plastic waste into structured carbon Joule heaters for ammonia decomposition (Fig. 2c), achieving near-zero-emission H_2_ production and offering a transformative step toward sustainable energy solutions. Yang *et al*. [61] designed a wood carbon monolith for a distributed electrified heating application in the catalyst-free hydrolysis of CH_4_ at 1150°C (Fig. 2d). Zhang *et al*. [25] employed carbon felt as a Joule-heating source (Fig. 2e) on which a hydrophilic, sulfonic-acid-functionalized covalent organic framework (COF–SO_3_H) was grown for a catalytic esterification reaction between carboxylic acids and alcohols, extending the electrothermal catalysis technology to the production of fine chemicals.

The programmability of electricity, coupled with the rapid temperature response to Joule heating, endows electrothermal catalysis with exceptional flexibility in heat management. Dong *et al*. [76] developed a pulsed heating and quenching technique by using a programmable electric current passing through carbon paper, the temperature of which was elevated to 2400 K within 0.02 s (Fig. 2f), leading to high selectivity for value-added C_2_ products through CH_4_ pyrolysis and good catalyst stability (preventing the sintering of the coated Ru nanoparticles) for stable NH_3_ synthesis under ambient pressure. Yu *et al*. adopted a similar strategy, enabling the *in situ* regeneration of catalysts from coking during the dry reforming of methane (DRM) [77] and steering the selectivity of CO_2_ hydrogenation [78].

These applications of carbon-based supports in electrothermal catalysis have achieved advanced features similar to those of metallic supports, such as fast heating and low energy consumption, even generating super-high temperatures that the latter cannot achieve. Despite the significant progress made, carbon materials as Joule-heating supports present two inherent drawbacks: weak mechanical strength and the risk of carbon oxidation in oxidative environments, restricting their potential application scope for electrothermal catalysis. Carbon materials with a high degree of graphitization would be preferable.

### Conductive ceramic supports

As inorganic materials, ceramics surpass carbon-based counterparts in mechanical robustness and chemical stability, offering resilience in high-temperature oxidative atmospheres. Some conductive ceramic materials, including perovskite, SiC and SiSiC, have been explored for electrothermal catalysis. Liu *et al*. [21] fabricated electrically conductive (La_0.80_Sr_0.20_)_0.95_FeO_3_/Gd_0.1_Ce_0.9_O_2_ composite ceramics coated with MnO_2_ catalysts (Fig. 2g) and achieved complete CO oxidation at a low temperature of 165°C using Joule heating. Renda *et al*. [79] utilized commercial SiC heating elements as the support and coated a Ni-based catalyst onto them for SRM and DRM reactions, realizing a reaction zone of 900°C. Moreover, the energy consumption for H_2_ production is comparable to that of modern electrolysis cells. Their subsequent work revealed that SiSiC foam exhibits electrified DRM activity that is higher than the SiC monolithic counterpart, approaching thermodynamic equilibrium conversion [80]. Meanwhile, the corresponding energy consumption for H_2_ production is close to theoretical limit values, equaling about a quarter of that obtained through microwave heating. Zheng *et al*. [30] proposed that the open-cell structure of SiSiC foams can largely enhance gas–solid mass transfer, overcoming the mass-transport limitations associated with the laminar flow in narrow tubes and the radial diffusion towards the tube walls (Fig. 2h). Therefore, a series of their works that focused on SiSiC foams as Joule-heating supports were reported to electrify H_2_ production through DRM, SRM and reverse water–gas shift [37,81,82,84]. Electrothermal catalysis using SiSiC foam enables selective and volumetric heating of the catalytic bed, providing a more uniform temperature distribution and thus resulting in significantly low specific energy demand. Huang *et al*. [83] also reported a highly efficient and stable electrothermal DRM process using a wash-coated Ni catalyst on SiC foam. So far, however, the number of conductive ceramics explored for electrothermal catalysis is small and more similar materials are needed for development.

**Figure 3.**
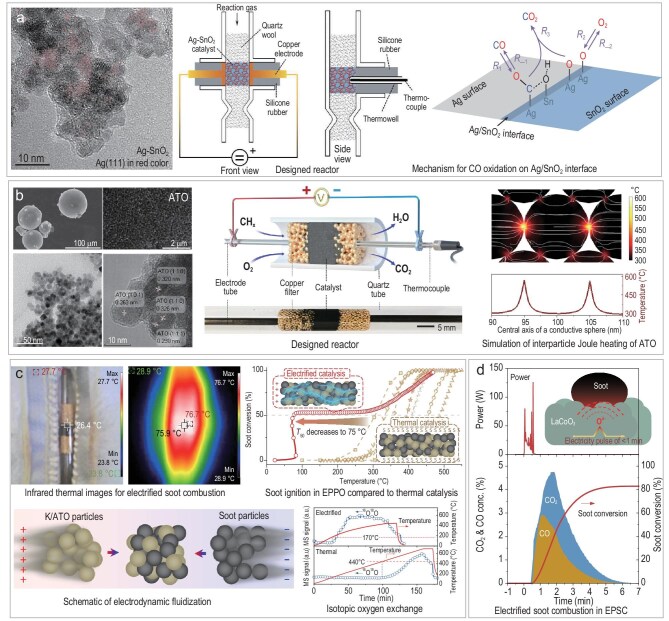
Electrothermal catalysis for oxidation removal of air pollutants using stacked interparticle conductive catalysts. (a) Ag–SnO_2_ catalyst for electrified oxidation of formaldehyde and CO. Adapted with permission from Ref. [92] (Copyright 2020, Royal Society of Chemistry). (b) ATO catalyst for electrified oxidation of hydrocarbons. Adapted with permission from Ref. [93] (Copyright 2023, Elsevier). (c) K/ATO catalyst for electrified soot combustion in electrically powered programmed oxidation (EPPO). Adapted with permission from Ref. [94] (Copyright 2021, Springer Nature). (d) LaCoO_3_ catalyst for electrified soot combustion in electricity-pulse-sparked-catalysis (EPSC). Adapted with permission from Ref. [95] (Copyright 2023, Elsevier).

### Monolithic Joule heating and electrical promotion

The advantages of electrothermal catalysis using monolithic conductive supports were elucidated in terms of monolithic Joule heating. These supports primarily provide rapid and flexible heating to the catalyst coating in intimate contact with them, resulting in uniform temperature distribution, enhanced energy utilization and compact reactor design (Table 2). Furthermore, swift adjustments of the Joule-heating modes, particularly in pulse forms, allow precise control over the reaction pathways under non-equilibrium conditions, improving selectivity of the desired products and catalyst stability, as successfully implemented in CH_4_ pyrolysis [76], NH_3_ synthesis [76], DRM [77] and CO_2_ hydrogenation [78].

Beyond Joule heating, electrical promotion directly acting on chemical processes was revealed in some studies by using monolithic conductive supports. In the studies of catalytic formaldehyde oxidation under the Joule heating of carbon-based supports, some highly reactive radicals of •O_2_^−^ and •OH were observed [23,73]. This readily brings to mind plasma catalysis, which features the generation of highly reactive radicals. However, the applied voltages in electrothermal catalysis are far lower than those of plasma catalysis, with large current flowing through the conductors, as listed in Table 1.

In addition to the formation of radicals, enhanced transformation of the lattice oxygen of catalysts to active oxygen species was observed under the Joule heating of monolithic supports, which is beneficial for oxidation reactions. Tao *et al*. [66] proposed a thermoelectric synergistic oxidation effect using a MnO_2_/TiO_2_–Ni foam catalyst, which significantly promotes the conversion of lattice oxygen to surface reactive oxygen for the oxidation of acetone. Dou *et al*. [22] found that the electric heating of Ni foam accelerates the reduction of Ni^2+^ to metallic Ni active sites, favoring CO_2_ methanation. Wang *et al*. [63] proposed that the electric heating of nano *δ*-MnO_2_/Ni foam catalyst facilitates the release of lattice oxygen, enhancing formaldehyde oxidation. Similarly, Zhou *et al*. [64] revealed that the electric heating of a CuO–ZnO/Ni foam catalyst promotes lattice oxygen release in the Ni skeleton and redox reaction of Cu/Zn species for H_2_ production from methanol decomposition. The electrically driven release of lattice oxygen was also reported in studies of electric-field-assisted catalysis [85,86]. Accordingly, the oxygen-containing radicals mentioned above may be transformed from the active oxygen species that are generated through the electrical release of lattice oxygen.

Electrical promotion can enhance intrinsic catalytic activity in ways that Joule heating cannot, as the latter is primarily limited to merely ameliorating the heating process for catalysis. However, when currents predominantly flow through monolithic conductive supports generating Joule heat, monolithic Joule heating becomes the dominant factor in electrothermal catalysis. Meanwhile, electrical promotion is constrained because currents tend to bypass catalyst coatings with minimal partial voltages across them. Despite high energy-utilization efficiencies, the results of some studies show that the improvement of Joule heating from monolithic supports on the catalytic activities is not significant compared with traditional thermal catalysis at the same global temperatures [19,57]. It can be speculated that, if the catalytic components that provide active sites are electrically conductive, which allows current to primarily flow through them, then electrical promotion could be fully exhibited, potentially leading to unexpected enhancement of the catalytic activity.

## ELECTROTHERMAL CATALYSIS USING INTERPARTICLE CONDUCTIVE CATALYSTS

### Stacked interparticle conductive catalysts

Electrothermal catalysis employing interparticle conductive catalysts, akin to conventional fixed-bed reactions with stacked catalyst particles, could amplify the electrical promotion. The adopted reactors were described as having a Probe–Bed–Probe configuration by McEwen *et al*. [87]. A series of modeling studies by Nikrityuk’s group [88–91] demonstrated the feasibility of internal heat generation using the Joule heat in fixed-bed reactors filled with metal particles. Our group has been devoted to exploring interparticle conductive catalysts featuring both catalytic activity and electrical conductivity. The initially selected catalyst was Ag–SnO_2_ (an electrical contact material) due to the exceptional catalytic oxidation activity of Ag [92]. Meanwhile, a cross-type reactor was fabricated to stack the Ag–SnO_2_ catalyst within, by which currents and reaction gases could orthogonally flow through the catalyst bed to trigger the oxidative removal of formaldehyde and CO as probe reactions (Fig. 3a). The performance was superior to that of the conventional thermal catalysis counterpart. Thereafter, we found that metal-oxide semiconductors—a class of unique charge-transport materials—are promising candidates. In this context, three metal oxides, including antimony–tin oxide (ATO), indium-tin oxide (ITO) and LaCoO_3_ perovskite, were selected as conductive catalysts. A corresponding homemade reactor was developed in which conductive catalysts stacked within a quartz tube were sandwiched by two copper filters connected to an adjustable direct-current power generator, allowing currents and reaction gases to parallelly flow through catalyst beds (Fig. 3b). Using this paradigm, we accomplished electrified hydrocarbon combustion by passing current through an ATO catalyst [93]. Compared with traditional thermal catalysis, the ignition temperatures were decreased by ∼100°C at the expense of a lower energy input by one order of magnitude, with high water tolerance.

A breakthrough in electrothermal catalysis was achieved when we used the metal-oxide semiconductors as conductive catalysts for the combustion of diesel soot—an important pollutant emitted from diesel engines—which is always slammed for its high ignition temperatures. Especially, there is a unique characteristic for electrothermal catalysis in soot combustion: the soot particles themselves are also electrically conductive. When current flows through a stacked mixture of conductive soot and a conductive catalyst such as ATO, ITO or LaCoO_3_ perovskite, the soot combustion is sparked at decreased ignition temperatures [94]. In detail, we designed an electrical control protocol, i.e. electrically powered programmed oxidation, through computer programming by which a linearly increasing electric power is applied to a catalyst–soot mixture to trigger soot combustion. The ignition temperature at which 50% of the soot (*T*_50_) is converted was decreased to <75°C when using the potassium-supported ATO (K/ATO) catalyst (Fig. 3c) [94,96]. This performance is far superior to that of the previously reported conventional thermal catalytic soot combustion—generally with *T*_50_ > 300°C. In a subsequent study [95], we devised another electrical control protocol, termed electricity-pulse-sparked-catalysis (EPSC), which involves applying an energy-intensive electricity pulse to catalyst–soot mixtures in a short time, sparking rapid soot combustion. With the use of a conductive LaCoO_3_ catalyst, the majority of soot is converted within <1 minute of electric pulse (Fig. 3d), resulting in extremely high reaction rates that overwhelm the results previously reported for catalytic soot combustion. Meanwhile, the energy efficiencies also exceed those of the previously reported plasma catalysis for soot combustion.

**Figure 4.**
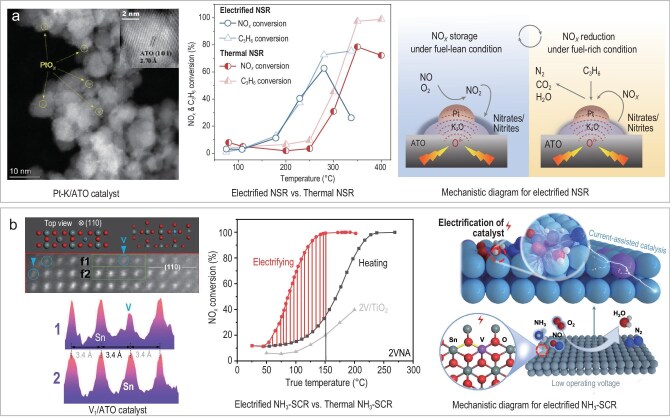
Electrothermal catalysis for NO*_x_* removal using ATO-based catalysts. (a) Pt–K/ATO catalysts for electrified NSR. Adapted with permission from Ref. [97] (Copyright 2023, American Chemical Society). (b) V_1_/ATO catalysts for electrified NH_3_–SCR. Adapted with permission from Ref. [26] (Copyright 2023, Springer Nature).

Metal-oxide semiconductor catalysts have also been investigated for the electrothermal catalytic removal of NO*_x_*, which is another air pollutant emitted from diesel engines. We put forward a conceptual process for the electrification of NO*_x_* storage reduction (NSR), which is a promising approach for removing the NO*_x_* emitted from lean-burned diesel engines [97]. In a typical NSR process, under fuel-lean conditions, NO is oxidized to NO_2_ and stored on NSR catalysts, while, under fuel-rich conditions, the stored NO*_x_* species are desorbed and reduced to N_2_ (Fig. 4a). The temporal flexibility of electricity especially benefits the cyclic NSR process. We prepared a Pt and K co-supported ATO conductive NSR catalyst (Pt–K/ATO) and applied low electrical powers (0.5–4 W) to electrify the NSR process (Fig. 4a). The ignition temperature for 10% NO*_x_* conversion was nearly 100°C lower than that for the traditional thermal counterpart and a 23% increase in the maximum energy efficiency was achieved by reducing the power in the fuel-lean period. Zheng *et al*. [26] adopted a similar paradigm of electrothermal catalysis for the selective catalytic reduction of NO*_x_* with ammonia (NH_3_–SCR) by using a fabricated monoatomic V-based ATO (V_1_/ATO) (Fig. 4b). This approach can completely convert NO*_x_* into N_2_ at ultra-low temperatures, offering high stability over 144 h of operation and reducing the energy consumption by >90%. Following an analogous strategy, they fabricated a Pd_1_–Ce_1_/ATO catalyst and achieved unprecedented activity for low-temperature CH_4_ combustion performance, with ∼80% CH_4_ conversion over 100 h of operation [98].

**Figure 5.**
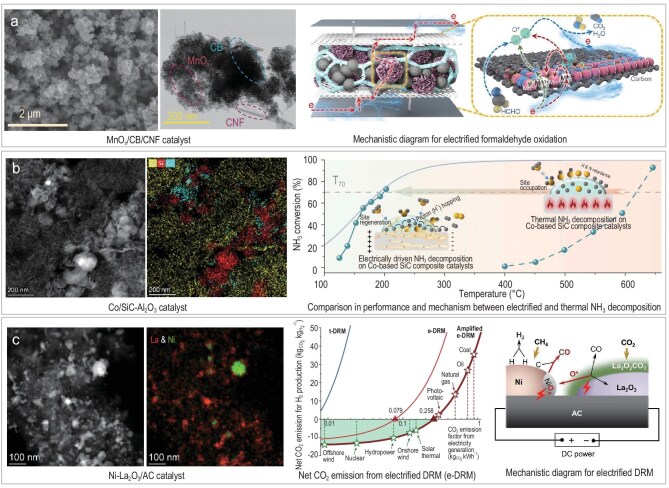
Electrothermal catalysis using conductive catalysts composited with carbon or SiC. (a) MnO*_x_*/CB/CNF catalyst for electrified formaldehyde oxidation. Adapted with permission from Ref. [24] (Copyright 2023, Elsevier). (b) Co/SiC–Al_2_O_3_ catalyst for electrified ammonia decomposition. Adapted with permission from Ref. [99] (Copyright 2025, Elsevier). (c) Ni–La_2_O_3_/AC catalyst for electrified DRM. Adapted with permission from Ref. [100] (Copyright 2025, AAAS).

Composite catalyst powders with common conductive materials, such as carbon and SiC, were also investigated for electrothermal catalysis. Fang *et al*. [24] intercalated both carbon black (CB) and MnO*_x_* nanoflowers into a 3D conductive aerogel with a cellulose nanofiber (CNF) skeleton for catalytic formaldehyde degradation (Fig. 5a). Applying a current to the MnO*_x_*/CB/CNF catalyst (termed ‘electro-injection’ in their work) enhances the formaldehyde-to-CO_2_ conversion efficiency with a 26.4% increase over that without electro-injection. Wang *et al*. [99] fabricated a Co/SiC–Al_2_O_3_ catalyst supported on SiC for electrically driven gaseous ammonia decomposition (Fig. 5b), which achieves 73% of ammonia conversion at ∼200°C, outperforming conventional thermal ammonia decomposition. Recently, we loaded both metallic Ni and La_2_O_3_ onto activated carbon (AC) and applied a current to the Ni–La_2_O_3_/AC catalyst to electrify DRM reactions (Fig. 5c) [100]. Electrified DRM outperforms conventional thermal DRM in CO_2_ and CH_4_ conversions and the H_2_/CO ratio, reaching thermodynamic equilibrium conversions and sustaining for ≥120 h. When the catalyst mass was scaled to the gram level, a landmark energy efficiency of 2.976 mmol kJ^−1^ was reached, surpassing previously reported values using plasma and pulsed laser strategies, thereby enabling the utilization of low-carbon electricity to achieve net CO_2_-negative emissions.

### Coated interparticle conductive catalysts

Different from monolithic conductive supports, interparticle conductive catalysts stacked within a reactor exhibit an unstable interparticle conductivity that varies depending on the different contact degrees between particles of varying sizes [30,31]. We have tried to address the issue through the reactor design and power control. In the reactor design, a coil spring is incorporated into the electrified reactor to compress the interparticle conductive catalysts, achieving consistent electrical resistance [101]. In power control, computer software was self-programmed to adjust the electric power output based on Joule’s law, by which the input voltage or current is determined by the real-time resistance and targeted power values, maintaining a stable electric power input [97].

**Figure 6.**
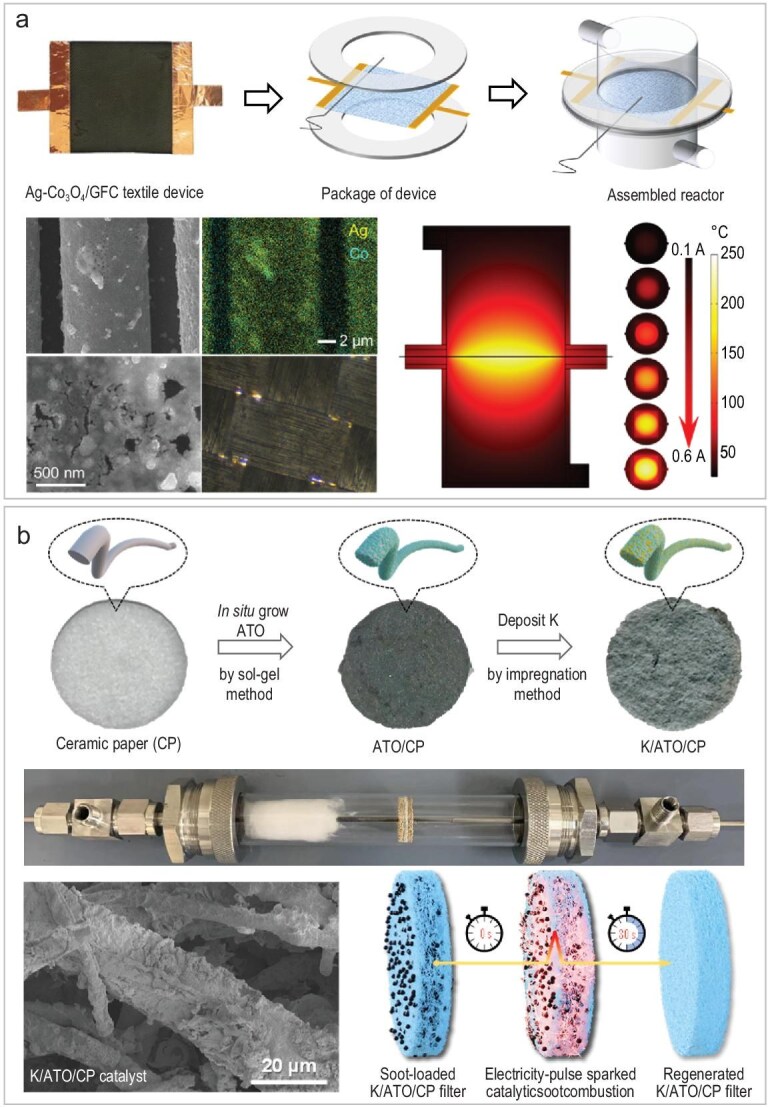
Coated interparticle conductive catalysts on electrically insulating monoliths for electrothermal catalysis. (a) Ag–Co_3_O_4_ coated on GFC for electrified oxidation of formaldehyde. Adapted with permission from Ref. [102] (Copyright 2021, Elsevier). (b) K/ATO coated on ceramic paper for electrified soot combustion in EPSC. Adapted with permission from Ref. [101] (Copyright 2024, Elsevier).

Apart from the reactor design and power control, a catalyst strategy—such as the deposition of interparticle conductive catalysts onto electrically insulating monoliths, generating a continuous conductive catalyst coating—is more radical for mitigating interparticle conductivity instability. When voltages are applied, the current can flow steadily through the coated catalyst particles, which are tightly arranged on the monolithic supports. In one of our works [102], a conductive Ag–Co_3_O_4_ layer was coated onto a glass-fiber cloth (GFC), capable of multifunctional air cleaning (Fig. 6a). Powered by low voltages (<20 V), the device exhibited a 3-fold formaldehyde conversion compared with the conventional thermal counterpart and energy savings of >90%. Another work from our group [101] employed aluminosilicate fiber-weaved ceramic paper as the support, on which a conductive K/ATO layer was coated for electrified soot combustion (Fig. 6b). The monolithic catalyst with a 3D-network structure has the potential for trapping soot particles. When the EPSC method that we developed was used, almost all of the loaded soot could be burned out within less than half a minute of electric pulse, achieving exceptional reaction rates and energy efficiencies. Furthermore, the high flame temperature caused by rapid soot combustion was localized within the monolithic catalyst, ensuring system safety. Zhang’s group [98] coated Pd_1_–Ce_1_/ATO catalysts onto SiC foam for electrothermal CH_4_ combustion, demonstrating significant potential for industrial applications.

### Interparticle Joule heating of electrothermal catalysis

Unlike monolithic conductive supports, which exhibit a relatively homogeneous distribution of electrical resistance, interparticle conductive catalysts feature high contact resistance between the catalyst particles. It can be predicted that, when current flows between conductive particles, this contact resistance results in localized Joule heating and the formation of hotspots (localized high-temperature zones). The phenomenon is termed ‘interparticle Joule heating’ here. As hotspots typically range in size from micrometers to nanometers, detecting them remains challenging with current temperature-measurement technologies, as conventional techniques such as infrared thermal imaging lack the spatial resolution required for their observation [24,26,93,94,99]. Nevertheless, interparticle Joule heating can be simulated by using numerical calculations. The modeling studies by Nikrityuk’s group [88–91] on conceptual reactors packed with mixed metal particles and nonconductive catalyst particles predicted that current flow creates a mostly uniform cross-sectional temperature, yet hotspots with temperature differences of ≤100°C can exist between particles. We also conducted a numerical simulation by using an electrothermal model to clearly describe the localized Joule heating between ATO nanospheres [93]. As shown in the right side of Fig. 3b, the electric current (in the form of streamlines) becomes concentrated to pass through the contact points between the ATO spheres, generating hotspots. A temperature profile along the horizontal center axis of the sphere displays that the temperature of the main body of the spheres is ∼300°C, while the temperature at the contact points reaches ∼600°C. Interparticle Joule heating, which arises on the contact surface between catalyst particles, can specifically direct its energy towards heating the active surface.

### Interparticle electrical promotion of electrothermal catalysis

Most studies on electrothermal catalysis using interparticle catalysts demonstrate superior performance compared with the conventional thermal counterpart at the same global temperatures, despite the hotspot effects discussed above. Mechanistically, this enhancement can be attributed to electrical promotion beyond Joule heating. Zheng *et al*. [26] suggested that both Joule heating and electrical promotion contribute to electrified NH_3_–SCR. Regardless of whether in stacked or coated architecture, the interparticle conductive catalysts were found to deliver significant electrical promotion for electrothermal catalysis in the aforementioned studies. In the study on the electrified oxidation of CO over the Ag–SnO_2_ catalyst [92], we deduced that an electric effect, namely the electron transport from Ag to SnO_2_, results in a negatively charged interface favoring O_2_ activation and suppressing CO poisoning. For electrified soot combustion using metal-oxide semiconductors as catalysts [94–96,101], we revealed two electrical promotion effects in addition to Joule heating. One effect is electrodynamic fluidization, which is exclusive to electrified catalytic soot combustion, in which the particles of the conductive catalyst and the conductive soot move under Coulomb forces in an electrical field (Fig. 3c). The relative movement between the two kinds of particles can substantially improve their contact for soot combustion—a typical solid (soot)–solid (catalyst)–gas (O_2_) reaction. The other effect is the electrically driven release of lattice oxygen, i.e. the electric power can drive the notable release of the lattice oxygen of metal-oxide semiconductors and the subsequent formation of surface-active oxygen species for oxidation. The effect was proved by using isotopic oxygen-exchange experiments, which showed that the electrically driven exchange between gaseous oxygen and lattice oxygen occurs at a much lower temperature compared with its thermally driven counterpart (lower-right corner of Fig. 3c). Furthermore, we demonstrated that the effect can be amplified via EPSC methods with energy-intensive electrical pulses (Fig. 3d) and used to accelerate the decomposition of surface sulfate to alleviate SO_2_ poisoning [101].

The electrically driven release of lattice oxygen has also been demonstrated as a key electrical promotion mechanism in NO*_x_* reduction. In our electrified NSR system using Pt–K/ATO catalysts [97], multiple steps of NSR—including NO adsorption, desorption and reduction—involve oxygen transfer and thereby are electrically enhanced (right side of Fig. 4a). Similarly, in the electrified NH_3_–SCR [26] and CH_4_ combustion [98] systems, Zhang’s group proposed an ‘electron scissors effect’, which can weaken the V–O, Pd–O and Ce–O chemical bonds and thus accelerate oxygen circulation (right side of Fig. 4b). These findings consolidate our results on the electrically driven release of lattice oxygen.

As mentioned above, the electrically driven release of lattice oxygen has likewise been reported in electrothermal catalysis using monolithic conductive supports [24,63,64,66] (see the ‘Monolithic Joule heating and electrical promotion’ section). When these supports (e.g. carbon, SiC) are integrated with catalytic components via interparticle configurations, the resulting conductive composite catalysts exhibit considerably amplified electrical promotion effects. For the MnO*_x_*/CB/CNF catalyst developed by Fang *et al*. [24], electron injection into the CB-intercalated structure leads to the migration of lattice oxygen and redistribution of electron density in the MnO*_x_*, accelerating the transformation among the O_2_, lattice oxygen and reactive oxygen species O* during the formaldehyde oxidation (Fig. 5a). In our electrified DRM system on the Ni–La_2_O_3_/AC catalyst [100], the electrically driven release of lattice oxygen bridges the synergy between the Ni and La_2_O_3_ for the CO_2_–CH_4_ redox cycles, not only enhancing the decomposition of the La_2_O_2_CO_3_ intermediates generated from the CO_2_ adsorption on the La_2_O_3_, but also promoting the reduction of the resultant NiO*_x_* species through the oxidation of CH_4_-dissociated coke (Fig. 5c).

In the study on electrically driven ammonia decomposition, Wang *et al*. [99] suggested a stimulated charge-migration effect by comparing the catalytic performance and reaction mechanism of two Co-based SiC composite catalysts, including a SiC-supported catalyst (Co/SiC–Al_2_O_3_) and a SiC-mediated catalyst (Co/Al_2_O_3_–SiC). The direct loading of Co species on highly conductive SiC in Co/SiC–Al_2_O_3_ facilitates the transportation of protons (H^+^) and electrons between the Co species and SiC under an electric field. Consequently, Co/SiC–Al_2_O_3_ exhibits more pronounced activity than Co/Al_2_O_3_–SiC. The electrical promotion mechanism involves stimulated proton hopping and electron migration, which promote the stepwise dehydrogenation of NH*_x_* and product desorption. This proton-hopping effect aligns with observations by Sekine’s group in electric-field-assisted catalysis [103].

## PROPOSING MONOLITHIC AND INTERPARTICLE ELECTROTHERMAL CATALYSIS

**Figure 7.**
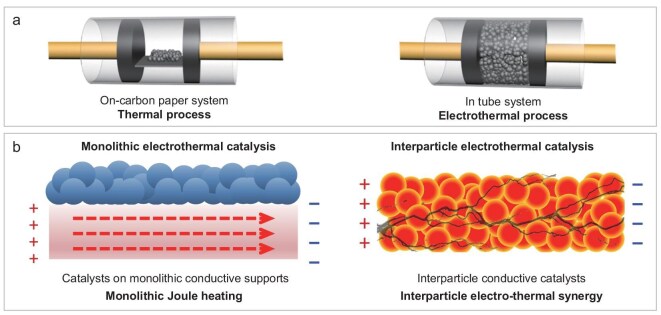
Monolithic and interparticle paradigms of electrothermal catalysis. (a) Schematics of reaction vessel for the conversion of amorphous carbon into flash graphene by using flash Joule heating. Adapted with permission from Ref. [104] (Copyright 2024, American Chemical Society). (b) Schematics of the monolithic and interparticle electrothermal catalysis that we proposed.

As discussed above, the review reveals two distinct catalyst architectures: catalysts on monolithic conductive supports versus interparticle conductive catalysts. This structural dichotomy dictates fundamentally different contributions from Joule-heating and electrical promotion mechanisms. Monolithic conductive supports predominantly exhibit a monolithic Joule-heating effect, whereas interparticle conductive catalysts deliver amplified electrical promotion synergizing with interparticle Joule heating. This structural dichotomy mirrors the phenomena observed in electrothermal graphene synthesis, in which analogous architecture-dependent heating mechanisms govern reaction kinetics. In a study on the conversion of amorphous carbon into flash graphene by using flash Joule heating, Eddy *et al*. [104] found that the process can be deconvoluted into a thermal process and an electric process, whose contributions are distinct in two designed reaction vessel architectures (Fig. 7a). In one reaction vessel, the current flows primarily through a carbon-paper platform that is analogous to the monolithic conductive supports on which the amorphous carbon is heated through Joule heating. In the other reaction vessel, the current flows through the amorphous carbon itself in the interparticle form. Compared with the purely thermal process in the former, an electrical promotion effect was found in the latter. A charge- and current-induced electric field inside the graphene precursor facilitates phase transition by lowering the activation energy of the reaction. Inspired by these mechanistic insights, we proposed a paradigm shift in electrothermal catalysis classification: monolithic electrothermal catalysis versus interparticle electrothermal catalysis (Fig. 7b).

In monolithic electrothermal catalysis, catalytic materials are coated on monolithic conductive supports—typically metallic, carbonaceous or ceramic structures. Under applied voltages, current flows predominantly through these supports. The resulting monolithic Joule heating dominates the catalytic process, as rapid thermal conduction from the support elevates active sites to reaction temperatures. Uniform current distribution generates homogeneous heat flux with minimal thermal gradients. However, electrical promotion remains constrained: current bypasses the catalysis layer, creating only minimal voltage drops across active sites, thereby limiting the enhancement of intrinsic catalytic activity.

**Figure 8.**
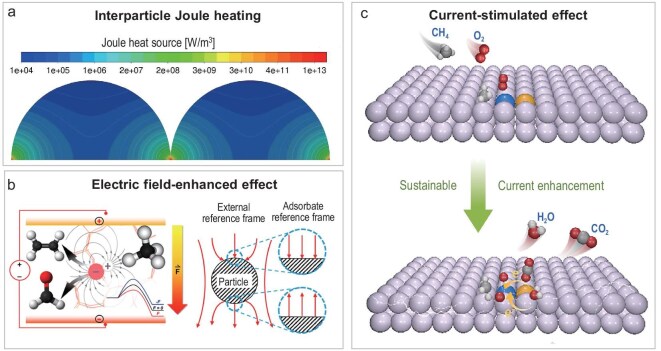
Underlying mechanisms of interparticle electrothermal catalysis. (a) Modeling results of interparticle Joule heating with hotspots between contact particles. Adapted with permission from Ref. [91] (Copyright 2022, ASME INTERNATIONAL). (b) Schematics of electric-field-enhanced effect on reaction (left side) and nonuniform surface electric fields on a catalyst particle embedded in a uniform electric field (right side). Adapted with permission from Ref. [87] (Copyright 2018, American Chemical Society). (c) Schematic of current-stimulated effect, exemplified by electrothermal catalytic methane combustion over a Pd_1_–Ce_1_/ATO catalyst. Adapted with permission from Ref. [98] (Copyright 2025, John Wiley & Sons).

In interparticle electrothermal catalysis, conductive catalyst particles form interconnected networks. When voltages are applied, the current flows through both particles and interparticle contacts, generating two synergistic effects. One is interparticle Joule heating featuring hotspots on contact interfaces, mainly governed by the contact radius and contact pressure between the particles (Fig. 8a). The effect provides the essential thermal energy (or working temperature) for the catalytic reaction. The other is interparticle electrical promotion, in which a flux of charge carriers modulates the electronic structures of the catalytic sites, thereby promoting the reactions. For example, the current weakens the metal–oxygen bond and thereby drives the release of lattice oxygen and surface oxygen cycling for redox reactions [26,94]. Besides, an electric field or current stimulates proton hopping for hydrogen production [99]. Compared with monolithic electrothermal catalysis, interparticle electrothermal catalysis maximizes electrical promotion, as almost all applied voltages act directly on the catalytic sites, remarkably enhancing intrinsic activity beyond Joule heating. Crucially, interparticle electrical promotion and interparticle Joule heating can operate synergistically to drive catalytic reactions.

The origin of the electrical promotion, whether from the electric field or the current, remains a subject of debate. The electric-field-enhanced effect, which was proposed in the studies on electric-field-assisted catalysis, in principle, alters the reaction thermodynamics by changing the electric moments (e.g. electric dipole moment, polarizability) of molecules or catalysts [87,105] (left side of Fig. 8b). Nevertheless, the attribution to the electrical promotion in interparticle electrothermal catalysis presents two uncertainties. First, despite a uniform electric field applied across the catalyst particles, the direction of the surface field experienced by a reactant molecule varies with its local position on the surface (right side of Fig. 8b). In the configuration, the effects of positive and negative fields may cancel each other out [87]. Second, due to the large current (amperes) that is characteristic of electrothermal catalysis (see the ‘Clarifying the scope of electrothermal catalysis’ section), any direct biasing effect of the voltage or electric field on the molecules or catalysts is likely negligible, consistent with the finding of Zhang’s group that, under an electric field without current, no activity enhancement was observed [98]. Therefore, it is highly likely that the electric-field-enhanced effect is not a dominant mechanism in interparticle electrothermal catalysis. In contrast, the current-stimulated effect is increasingly supported by experimental evidence. It was described as ‘electrical release of lattice oxygen’ [94], ‘electron scissor effect’ [26], ‘stimulated proton hopping’ [99] or ‘enhanced electron transfer’ [98] (Fig. 8c). Likewise, the flow direction of the current relative to the catalyst particle surface varies, which requires further consideration.

## PROSPECT AND CHALLENGES

### Prospect

The paradigm shift from monolithic to interparticle architectures promises transformative advances in electrothermal catalysis:

- The pronounced enhancement of intrinsic catalytic activity through sufficient electrical promotion enables lower reaction temperatures and improved energy efficiency. This efficiency gain directly reduces the carbon footprint across the full life cycle. In principle, when powered by fully renewable electricity and assessed within appropriate boundaries of life-cycle assessment (LCA), electrified CO_2_-utilization processes are anticipated to achieve carbon-neutral or even net-carbon-negative emissions [106,107].
- The interparticle architecture of catalysts offers compelling advantages for rapid industrial deployment within existing manufacturing frameworks. Its granular form facilitates integration into conventional fixed-bed reactors, while low-voltage electrification interfaces with established automation platforms. This compatibility reduces implementation cycles and requires minimal capital expenditure.
- The electrical promotion of interparticle electrothermal catalysis cannot be adequately explained by using conventional thermal catalysis frameworks. Fundamental investigation into the unique chemical phenomena arising from electrothermal synergies promises to establish a new interdisciplinary frontier in catalysis science.

### Challenges

The ongoing paradigm shift toward interparticle electrothermal catalysis faces critical technological and scientific barriers that demand concerted multidisciplinary efforts:

- Industrial application: despite its industrial attractiveness, the real-world application of interparticle electrothermal catalysis faces key challenges. First, interparticle conductive catalysts such as ATO, ITO and perovskite typically exhibit a low specific surface area, limiting their activity. Advanced synthesis technologies such as the template-assisted hydrothermal method [108] are preferable. Second, the unstable interparticle contacts and the resulting hotspots raise significant safety and stability concerns. Based on investigations on the evolved contact resistance, current distribution and hotspot intensity over time, these concerns can be mitigated by designing catalysts that are tolerant of high temperatures [109], designing the coatings on monoliths, assembling robust reactors, implementing precise thermometry and prediction, and applying computer-aided feedback power control. Third, expanding applications to fluidized bed reactors presents the challenge of low conductivity between colliding particles, which requires innovations in catalyst design and reactor configurations. Fourth, the on-site utilization of renewables with intermittency and fluctuation challenges the stable operation of interparticle electrothermal catalysis. This can be addressed by implementing advanced thermal management strategies, integrating them with energy-storage systems and designing catalysts for wide temperature tolerance [109]. Finally, a convincing demonstration of industrial potential will necessitate large-scale, long-term testing combined with numerical simulation and LCA to evaluate both technical viability and sustainability.
- Catalyst temperature measurement: the catalyst temperature serves as a critical metric for benchmarking interparticle electrothermal catalysis against conventional thermal processes. The superior reaction kinetics observed at identical temperatures provide evidence of non-thermal electrical promotion beyond Joule-heating effects. At the microscale, catalyst temperature distributions exhibit spatial heterogeneity arising from interparticle Joule heating. Localized Joule heating with hotspots occurring between catalyst particles has been predicted through numerical simulation [88,89,93]. However, current infrared thermal imaging techniques, which have spatial resolution at the micron level, fail to capture these hotspots in nanosized zones. Recently, Tian *et al*. [110] developed a confocal two-photon microscopy-based approach to measure the spatiotemporal variation in temperature within individual catalysts during catalytic reactions. However, applying this methodology to interparticle electrothermal catalysis presents considerable challenges due to its operational complexity and high equipment dependency.
- *In situ* characterization: *in situ* techniques are indispensable for elucidating the reaction mechanisms in electrothermal catalysis. Our group developed an *in situ* Raman spectroscopy platform to probe catalyst structural evolution during electrified soot combustion, which revealed electrically driven lattice oxygen release as a key promotion mechanism [94]. Zheng *et al*. developed *in situ* diffuse reflectance infrared Fourier transform spectroscopy and *in situ* X-ray photoelectron spectroscopy techniques, leading to a similar finding on the electrical promotion effect [26]. However, designing specialized *in situ* reaction cells remains challenging, requiring both: (a) current delivery through catalyst beds under operational conditions and (b) compatibility with characterization instrument geometries.
- Theoretical calculation: quantum chemistry calculations have been employed to elucidate the electric-field effects in electrothermal catalysis [23,26], paralleling methodologies in electric-field-assisted catalysis studies [105]. These simulations consistently predict field-induced promotion mechanisms, including increased electron density at active sites and weakened metal–oxygen bonds. However, some discrepancies undermine their reliability. First, the electric-field intensities required to elicit significant effects in computational models (in the order of V/Å) far exceed experimental values (in the order of V/cm) by seven to eight orders of magnitude. Second, the orientation of the electric field relative to the catalyst surface exerts divergent or even reversed effects on computational outcomes. Lastly, models neglect electrical current—a dominant factor in electrothermal systems in which the carrier flux may override static field effects.
- Decoupling Joule-heating and electrical promotion contributions: both effects simultaneously arise when current flows through interparticle conductive catalysts, blurring the line between them. Generally, the electrical promotion contribution primarily accounts for the performance enhancements relative to conventional thermal catalysis at the same global temperatures. However, this attribution is complicated by the presence of microscopic hotspots, the contribution of which to the activity enhancement cannot be entirely ruled out. Another feasible strategy involves implementing targeted thermal management (e.g. reactor cryocooling) to enhance heat dissipation [94,95,99]. Weakening the influence of Joule heating makes it possible to isolate and confirm the contribution of electrical promotion. Fully decoupling electrical promotion from Joule heating necessitates a multi-technique approach, such as high-spatial-resolution operando thermometry, heat/energy-balance normalization to the effective active volume and coupled electrothermal modeling to bound hotspot contributions.
- Disentangling field versus current contributions to electrical promotion: as discussed in the ‘Interparticle electrical promotion of electrothermal catalysis’ section, the direct contribution of the electric field to electrical promotion is likely negligible due to the nonuniform surface field and the minimal effective bias voltage on molecules and catalysts. Still, this speculation requires more and stronger experimental and theoretical evidence. Considering the large current (amperes) that is characteristic of interparticle electrothermal catalysis (see the ‘Clarifying the scope of electrothermal catalysis’ section), the primary driver of the electrical promotion is most likely the current rather than the field, which has been confirmed by several studies [26,98,99]. From electronic-level insights, resolving the mechanistic linkage between carrier drift (physical current) and charge transfer (chemical processes) demands synergistic collaboration between physicists and chemists.

## CONCLUSIONS

Amidst the global pursuit of carbon neutrality, electrified catalysis technologies are rapidly emerging to harness renewable electricity for chemical processes, thereby reducing the dependence on fossil fuels and decreasing the carbon footprint. Amongst them, electrothermal catalysis utilizing catalyst Joule heating stands out due to its efficient and flexible heat generation, compact reactor design, rapid startup/shutdown capability, high energy utilization and exceptional catalytic efficiency. To distinguish it from other electrified catalysis, we define its scope by clarifying unique characteristics: conductive catalysts, Joule heating induced by high current (amperes) under low voltage (several to dozens of volts) and independence from external heating. Subsequently, the present review identifies two catalyst architectures for electrothermal catalysis: catalysts on conductive monolithic supports and conductive interparticle catalysts, which deliver fundamentally different contributions from Joule-heating and electrical promotion mechanisms, and are defined as monolithic and interparticle paradigms, respectively. In the monolithic case, Joule heating arising within conductive supports (composed of metal, carbon or ceramics) dominates catalytic systems, in which efficient thermal transfer to catalyst coating enables rapid, compact and spatially uniform heating under geometric confinement. In interparticle systems, current traversing conductive catalyst particles and their contact points induces localized Joule heating and amplified electrical promotion, exemplified by electrically driven lattice oxygen release, electrically induced electron transfer or electrically stimulated proton hopping. The electrothermal synergy typically enables unexpected catalyst performance, achieving remarkably lower reaction temperatures and substantially higher energy efficiency. Therefore, the shift from monolithic to interparticle paradigms in electrothermal catalysis would establish fully electrified reaction systems, thus allowing the complete utilization of renewable electricity for low-carbon and potentially negative-carbon chemical processes.
